# Oxidized high‐density lipoprotein enhances endocrine disorders and ovarian damage in rats

**DOI:** 10.1111/jcmm.16197

**Published:** 2021-08-04

**Authors:** Lu Wang, Hongjuan Li, Xiaoke Tang, Yupei Yang, Yuancui Xiang, Hui Zhang, Yali Wang

**Affiliations:** ^1^ Department of Obstetrics and Gynecology Zhengzhou Central Hospital Affiliated Hospital to Zhengzhou University Zhengzhou China

**Keywords:** FOS, Granulosa cells, micro‐RNA‐34a, Oxidized high‐density lipoprotein, p65, Polycystic ovary syndrome

## Abstract

Previous findings have highlighted the association between oxidized high‐density lipoprotein (ox‐HDL) and polycystic ovary syndrome (PCOS) development; however, the underlying mechanism remains unclear. Under such context, the present study aimed to investigate the mechanism underlying the involvement of ox‐HDL in PCOS in relation to the p65/micro‐RNA‐34a (miR‐34a)/FOS axis. PCOS rat models were established with the injection of dehydroepiandrosterone (6 mg/100 g body weight). Both PCOS‐modelled rats and granulosa cells (GCs) were received treatment with ox‐HDL in order to identify its role in PCOS. Next, apoptosis and viability of GCs were detected with the application of TdT‐mediated dUTP Nick‐End Labeling and flow cytometry and Cell counting kit‐8, respectively. A series of assays were performed to determine the interaction among ox‐HDL, p65, miR‐34a, FOS and nuclear factor‐κB (NF‐κB). The results revealed high expression of ox‐HDL in PCOS, and enhanced endocrine disorders and ovarian damage in rats. ox‐HDL promoted apoptosis of GCs and decreased its viability. ox‐HDL activated NF‐κB pathway and induced p65 phosphorylation to promote miR‐34a expression. miR‐34a targeted and inhibited FOS expression. In conclusion, our findings suggested that ox‐HDL promoted the activation of p65 and transcription of miR‐34a, which stimulated apoptosis of GCs and inhibited expression of FOS, resulting in the overall acceleration of PCOS development.

## 1 INTRODUCTION

Polycystic ovary syndrome (PCOS) is a highly prevalent endocrine disease affecting 19.9% of women of reproductive age, and the prevalence of which ranges between 6% and 15% based on the diagnostic criteria applied.^1^, ^2^ For women with clomiphene citrate‐resistant PCOS, the application of medical ovulation‐induction agents may be time‐consuming and could result in adverse events like multiple pregnancies and cycle cancellation due to an excessive response, while risks like complications from anaesthesia, infection, and adhesions may often occur during or after surgery.^3^ Recently, correlations were found between enhanced apoptosis of granulosa cells (GCs) and enhancement of PCOS development.^4^ Therefore, identification of novel molecular mechanism mediating GC apoptosis in PCOS could provide further insight regarding PCOS development.

One of the serious complications of PCOS involves abnormalities related to metabolism of lipoproteins, including cholesterol, TG, low‐density lipoprotein (LDL) and high‐density lipoprotein (HDL).^5^ Formation of ox‐HDL induces the proliferation of vascular smooth muscle cells, resulting in the dysfunction of endothelial cells (ECs) and endothelial progenitor cells (EPC), while promoting oxidative stress and apoptosis of monocytes/macrophages.^6^ However, there is lack of reports elucidating the role of ox‐HDL in PCOS. Moreover, it has been found that a decreased amount of the anti‐atherosclerotic HDL particles was observed in hepatocytes, which is partly attributed to nuclear factor‐κB (NF‐κB) activation and thus recruitment of p65/50 proteins to NF‐κB binding sites, which is indicative of a potential interaction between HDL and NF‐κB p65.^7^ A previous study found that p65 can serve as a direct transcription factor to up‐regulate micro‐RNA (miR)‐34a.^8^ Moreover, miR‐34a promoted the apoptosis of GCs and inhibited the normal function of ovarian epidermal cells.^9^ However, the effect of miR‐34a on PCOS is scarcely understood. A prior study revealed the presence of decreased levels of FOS protein level in placental villi of the PCOS rats^10^ and in ovarian tissues of PCOS patients.^11^ An analysis of human genomic sequences from the tentative promoter of miR‐34a gene showed the presence of NF‐κB, c‐FOS and p53 response elements.^12^ On the basis of these prior findings, we conducted the present study to investigate whether ox‐HDL affected PCOS and the underlying mechanism involved in the regulatory mechanism of p65/miR‐34a and FOS in PCOS rat model and GCs treated with ox‐HDL.

## 2 METHODS

### 2.1 Ethical approval

The study protocol was approved by Ethics Committee of Zhengzhou Central Hospital Affiliated Hospital to Zhengzhou University. Participants were informed of the purpose of the study and signed informed consent prior to their enrolment. Animal experiments were performed in accordance with *Guide for the Care and Use of Laboratory Animals* published by the National Institutes of Health with ratification of the Animal Ethics Committee of Zhengzhou Central Hospital Affiliated Hospital to Zhengzhou University (Approval No. 201 901 016).

### 2.2 Patient enrolment

Thirty‐two female PCOS patients were selected for the study, while 35 healthy individuals served as controls. PCOS diagnosis was made based on the Rotterdam diagnostic criteria of PCOS (Rott‐2003)^13^: ovulation reduction, biochemical and/or clinical hyperandrogenism. The polycystic ovaries were examined with the use of ultrasonography to rule out other causes, such as congenital adrenal hyperplasia, androgen‐secreting cancer and Cushing's syndrome. The healthy individuals were examined by ultrasound to ensure that they had regular menstrual cycles, normal androgen levels and ovarian morphology, with the absence of hyperandrogenismas.

The patients underwent fasting for 12 h on the third to fifth day of the menstrual cycle, and the blood samples obtained from women with normal menstrual cycle were placed on ice. After 12 h of fasting, samples were obtained from the remaining tests in the morning, with centrifugation carried out immediately at 4°C for 15 min at 1900 g within 2 h. The concentration of serum ox‐HDL was detected by Enzyme‐linked immunosorbent assay (ELISA) Kit (Cell Biolabs, San Diego, CA, USA).

### 2.3 Induction of PCOS rat model

Female Sprague‐Dawley (SD) rats aged 21 days were fed adaptively for 2 days, and randomly assigned into the normal group and the PCOS group. For establishment of PCOS model, rats received continuous subcutaneous injection with dehydroepiandrosterone (DHEA) (6 mg/100 g body weight) for 30 days, while the normal group only received injection with 0.2 mL normal saline for injection during this period. On the 30th day of DHEA injection, the rat model found without ovulation and appearance of keratinocytes in the vaginal smear was indicative of successful rat model establishment. Then, the rats underwent fasting for 12 h and weighed, and the ovarian tissues were removed.

For rats treated with ox‐HDL (Shanghai AngYu Biotechnology Co., Ltd., Shanghai, China), ox‐HDL was dissolved in chitosan hydrogel (Human; Millipore, Billerica, MA, USA), and rats were injected intraperitoneally with ox‐HDL (10 mg/kg body weight) while the control group was injected with the same amount of vector (chitosan hydrogel) after 5 days of DHEA injection, once every 5 days. Next, the rats were euthanized by cervical dislocation, and the ovaries were extracted.

According to the above treatment, normal rats were only injected with normal saline or injected with normal saline and ox‐HDL (10 mg/kg; every 5 days), whereas PCOS‐modelled rats induced by DHEA were left untreated or injected with ox‐HDL (10 rats/treatment).

For grouping, rats were either left without treatment (PCOS + ox‐HDL group) or injected with LV5‐GFP‐short hairpin RNA (sh)‐negative control (NC) lentivirus + NC antagomir (PCOS + ox‐HDL + sh‐NC + NC antagomir group), LV5‐GFP‐sh‐p65 lentivirus (PCOS + ox‐HDL + sh‐p65 group), miR‐34a antagomir (PCOS + ox‐HDL + miR‐34a antagomir group) or pSIH1‐H1‐copGFP‐overexpression (oe)‐FOS lentivirus (PCOS + ox‐HDL + oe‐FOS group) via a tail vein prior to treatment of PCOS + ox‐HDL. All lentiviruses and antagomir were purchased from Gene Pharma (Shanghai, China).

The packed virus and the target vectors were cotransfected into HEK293T cells, and the supernatant was harvested 48 h after cell culture. The supernatant containing virus particles was obtained after filtration and centrifugation to detect the virus titre. The virus at logarithmic growth phase was condensed into a virus titre of 1 × 10^9^ TU/mL. As per the animal operation manual of antagomir and lentivirus products, antagomir was injected into rats at 80 mg/kg, and lentiviral particles were injected at 1 × 10^8^ TU/mL per rat.

### 2.4 Haematoxylin and eosin (HE) staining

Part of the paraffin‐embedded ovary tissues were cut into 4‐μm sections, dewaxed and hydrated before HE staining. After gradient ethanol treatment, the stained part was cleared with xylene and fixed in neutral resin. The morphological structure of ovarian follicles was observed under a light microscope.

### 2.5 TdT‐mediated dUTP Nick‐End Labeling (TUNEL) staining

As per the instructions provided by TUNEL detection kit (HRP‐DAB; E‐CK‐A331, Wuhan Elabscience Biotechnology Co., Ltd., Wuhan, China), the ovaries of rats were subjected to TUNEL staining. Apoptosis index (AI) = (number of apoptotic cells/total cells) × 100%.

### 2.6 ELISA

According to the manufacturer's instructions, the serum hormone levels of follicle‐stimulating hormone (FSH) (E‐EL‐R0391c), luteinizing hormone (LH) (E‐EL‐R0026c), testosterone (T) (E‐EL‐0072c) and estradiol (E_2_) (E‐EL‐0065c) were detected by ELISA kit (Elabscience, Wuhan, Hubei, China). The concentration of serum ox‐HDL was measured by commercial ELISA Kit (Cell Biolabs, San Diego, CA, USA). After the GCs were treated with ox‐HDL, the contents of cytokines interleukin (IL)‐1β (E‐EL‐R0012c), TNFα (E‐EL‐R2856c) and IL‐6 (E‐EL‐M0044c) in the cell supernatant were detected by ELISA kit (Elabscience, Wuhan). The experimental procedures were strictly in accordance with the instructions of the kits. The absorbance at 450 nm was measured and recorded.

### 2.7 Isolation and culture of ovarian GCs

GC was isolated from 21‐day‐old immature female SD rats. The ovaries were obtained on a sterile workbench and moved to pre‐cooled sterile PBS. The isolated ovaries were transferred to a preheated medium TCM199 (Gibco, Grand Island, New York) containing 0.1% bovine serum albumin (BSA), 100 IU/mL penicillin and 100 μg/mL streptomycin. All surrounding tissues were removed on the ovaries. The ovaries were repeatedly punctured with pin 25 in a 100 mm Falcon dish to release GCs. After filtration through a 200‐mesh cell sieve, the GC suspension underwent centrifugation at 200 g for 5 min, followed by resuspension in DMEM/F12 medium (Gibco) containing 0.1% BSA, 100 IU/mL penicillin and 100 μg/mL streptomycin, and finally incubated in 5% CO_2_ at 37℃. After incubation for 24 h, the culture medium was renewed and the adhered cells were used for subsequent experiments.

### 2.8 Transfection of GCs

The logarithmic growth phase cells were inoculated into 6‐well plates (3 × 10^5^ cells/well). When the cell confluence reached 70%‐80%, the cells were transfected according to Lipofectamine 3000 instructions (L3000008, Invitrogen, Carlsbad, CA, USA). The lentiviral vectors were transduced into cells at 2 μg per well, and miR‐34a inhibitor and mimic were transfected into cells at 10 nmol per well. The cells were transfected for 24 h. For the cells treated with ox‐HDL, the cells were cultured ox‐HDL at different concentration (0, 20, 50 and 100 μg/mL) for 24 h. Unless specified below, the concentration of ox‐HDL was 100 μg/mL.

The cells were treated with 0, 20, 50 or 100 μg/mL ox‐HDL, respectively, as the ox‐HDL group. In addition, cells were untreated (NC group), or cells were only transfected with empty vectors (NC + NC mimic + sh‐NC + oe‐NC group), or cells were transfected with empty vectors (ox‐HDL + NC mimic + sh‐NC group), with sh‐p65 (ox‐HDL + sh‐p65 group), with miR‐34a inhibitor (ox‐HDL + miR‐34a inhibitor group) or with oe‐FOS (ox‐HDL + oe‐FOS group) before treatment with 100 μg/mL ox‐HDL). All above plasmids were from Shanghai GenePharma Co., Ltd. (Shanghai, China).

### 2.9 Cell counting kit (CCK)‐8 assay

A freshly prepared preheated mixture (100 μL) containing DMEM/F12 and 10% CCK‐8 (Beyotime biotechnology, Shanghai, China) was added to GCs in each well. Then, the mixture was changed to a 96‐well plate. The plate was incubated at 37℃ for 2 h, and the optical density (OD) was measured at wavelength of 450 nm in each group.

### 2.10 Flow cytometry

After PBS washing, the collected cells were resuspended in 100 μL binding buffer, cultured with 2 μL Annexin V‐fluorescein isothiocyanate (FITC) (20 μg/mL) on ice for 15 min and then transferred to the flow tube. Cells were added with 300 μL PBS, and each sample was supplemented with 1 μL propidium iodide (PI; 50 μg/mL). Apoptosis was detected within 30 min.

### 2.11 Dual luciferase reporter assay

Cells were seeded into 24‐well plates at a density of 2 × 10^5^ cells/well. And 24 h later, the cells were transfected with pNF‐κB‐TA‐luc (Beyotime) and marine luciferase pRLSV (Promega, Madison, Wisconsin, USA) according to Lipofectamine 3000 instructions (L3000008, Invitrogen) as internal standardized control. Twenty‐four h prior to transfection, cells were treated with ox‐HDL or lentivirus for 24 h, and then cotransfected with pNF‐κB‐TA‐luc and pRLSV. And 48 h after transfection, the luciferase activity was detected on a Luminometer TD‐20/20 detector (E5311; Promega) with the use of the dual luciferase reporter assay system kit (Promega).

The promoter region of FOS mRNA 3'UTR containing miR‐34a binding site was synthesized. The FOS 3'UTR‐wild‐type (WT) and FOS 3'UTR‐mutant (MUT) plasmids were constructed and introduced into pGL3‐REPORT™ miRNA expression reporter gene vector system (Promega). The above plasmids were then cotransfected with miR‐34a mimic and mimic NC, respectively. The luciferase activity was measured on Luminometer TD‐20/20 detector (E5311; Promega) with dual luciferase reporter assay system kit (Promega).

### 2.12 Immunofluorescence

GCs were fixed in PBS with 2% paraformaldehyde, and fixed on the cover glass coated with poly‐L‐lysine at room temperature for 15 min, and then blocked overnight with PBS containing 10% FBS, 1% BSA, 0.05% Triton X‐100 and 2 mM EDTA. Next, GCs were stained with Rabbit anti‐NF‐κB p65 (Cell Signaling Technology, Beverly, MA, USA, 8242S; 1:100) for 2 h. immunoglobulin G (IgG)‐AF594 (Jackson Immune Research) was used to detect the antibody binding. DNA was stained with Draq5 (Cell Signaling Technology). The laser confocal experiment was carried under a Nikon TE2000‐U inverted microscope.

### 2.13 Immunohistochemistry

The ovarian tissue sections were dewaxed and rehydrated, followed by treatment with antigen recovery solution (pH 6.0 EDTA citrate buffer; Servicebio, G1202, China), and sealing with BSA (Servicebio, G5001, China). The sections then underwent incubation overnight with primary antibody (anti‐FOS, ab190289, Abcam, Cambridge, UK) at 4℃. After incubation with the secondary Goat anti‐rabbit IgG for 50 min, the sections were fixed. The sections were then imaged with Olympus BX51 microscope and DP73 CCD Olympus imaging system (Olympus Corporation, Tokyo, Japan).

### 2.14 Reverse transcription quantitative polymerase chain reaction (RT‐qPCR)

Trizol (Thermo Fisher Scientific, Rockford, IL, USA) was used to extract total RNA. Complementary DNA (cDNA) was synthesized using a reverse transcription kit (RR047A, Takara, Tokyo, Japan). For miRNA, the PolyA tailing assay kit (b532451, Sangon Biotech, Shanghai, China) was used to obtain the cDNA of miRNA containing PolyA tail. Then, qPCR was performed using ABI7500 qPCR instrument (ABI, Foster City, CA, USA) accordingly to TaqMan Gene Expression Assays protocol (Applied Biosystems, FOSter City, CA, USA). The 2^‐ΔΔCt^ represented the relative expression of the target gene which was normalized to U6 and glyceraldehyde‐3‐phosphate dehydrogenase (GAPDH) mRNA levels. The primers used are shown in Table 1.

**TABLE 1:** Primer sequences

Targets	Primer sequences (5’‐3’)
miR‐34a	F: 5’‐ATGGTTCGTGGGTGGCAGTGTCTTAGCTGG‐3’
IL‐1β	F: 5’‐CTCCATGAGCTTTGTACAAAG‐3’
IL‐6	R: 5’‐TGCTGATGTACCAGTTGGGG‐3’
	F: 5’‐AAGAAAGACAAAGCCAGAGTC‐3’
TNF‐α	R: 5’‐CACAAACTGATATGCTTAGGC‐3’
	F: 5’‐AAATGGGCTCCCTCTCATCAGTTC‐3’
FOS	R: 5’‐TCTGCTTGGTGGTTTGCTACGAC‐3’
	F: 5’‐CCAGAGCCAGGCCTAGAAGA‐3’
U6	R: 5’‐CTGCGAACCCTTCGTTTTTC‐3’
	F: 5’‐CTCGCTTCGGCAGCACA‐3’
GAPDH	R: 5’‐ACGCTTCACGAATTTGCGT‐3’
	F: 5’‐GACAACTTTGGCATCGTGGAA‐3’
	R: 5’‐CACAGTCTTCTGAGTGGCAGTGA‐3’

### 2.15 Western blot analysis

Total protein was extracted with the use of radio‐Immunoprecipitation Assay lysate buffer (R0010; Beijing Solarbio Science & Technology Co., Ltd. Beijing, China). Protein concentration of each sample was determined by using a BCA kit (GBCBIO Technologies, Guangzhou, Guangdong, China). The protein was separated by polyacrylamide gel electrophoresis, transferred to a polyvinylidene fluoride membrane (Millipore) and sealed with 5% BSA at room temperature for 1 h. Primary rabbit antibodies (1:1000) from Abcam to cleaved‐Caspase3 (ab49822), Caspase3 (ab13847), B cell lymphoma‐2 (Bcl‐2; ab196495), Bcl‐2‐associated X (Bax; ab32503), FOS (ab190289) and p65 (ab16502) as well as primary rabbit antibody to p‐p65 (Ser536) (1:1000; 3033, Cell Signaling Technology) were added to the membrane and incubated overnight. The following day, the membrane was incubated with goat anti‐rabbit IgG (ab97051, 1:2000, Abcam) at room temperature. The immunocomplexes on the membrane were visualized using enhanced chemiluminescence reagent and imaged using Image Quant LAS 4000C gel imager (GE, General Electric Company, Schenectady, NY, USA). With rabbit anti‐β‐actin (1:3000; Abcam, ab8227) serving as an internal reference, the grey value of each band was analysed by gel image analysis software Image J.

### 2.16 Statistical analysis

All data were processed using SPSS 21.0 statistical software (IBM Corp. Armonk, NY, USA). Measurement data were expressed as mean ± standard deviation. Unpaired t test was used for data comparison between two groups and one‐way ANOVA was used for data comparison among multiple groups, followed by Tukey's post hoc test. Data comparison among multiple groups at different time points was conducted by repeated‐measures ANOVA, followed by Bonferroni post hoc test. The difference was statistically significant at *P* < .05.

## 3 RESULTS

### 3.1 High expression of ox‐HDL in PCOS patients and PCOS‐modelled rats

**FIGURE 1:**
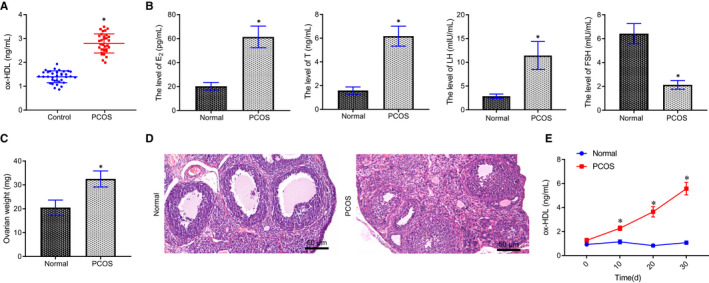
Expression of ox‐HDL is high in PCOS patients and PCOS‐modelled rats. A, The level of ox‐HDL in serum of PCOS patients (n = 32) and healthy controls (n = 35) detected by ELISA, * *P* < .05 compared with the control group. B, Serum E_2_, T, LH and FSH levels in PCOS and normal rats detected by ELISA, * *P* < .05 compared with the normal group. C, The weight of ovaries, * *P* < .05 compared with the normal group. D, The histopathological changes of ovarian tissue in the two groups observed by using HE staining (× 200). E, The levels of ox‐HDL in serum of rats in the two groups detected by ELISA at the 10th, 20th and 30th day after DHEA treatment, * *P* < .05 compared with the normal group. Measurement data were expressed as mean ± standard deviation. Unpaired t test was used for data comparison between two groups and data comparison at different time points was conducted by repeated measures ANOVA, followed by Bonferroni post hoc test. The experiment was repeated 3 times

The level of ox‐HDL in PCOS patients was significantly higher than that in the healthy controls (Figure 1A). After 20‐day observation of PCOS modelling, the normal oestrous cycle was maintained in the normal rats, while the PCOS‐modelled rats exhibited irregular female cycle of anovulation. Compared with the normal rats, the E_2_, T, and LH levels were increased, while FSH level was decreased in the PCOS‐modelled rats (Figure 1B). The ovarian weight of PCOS rats was significantly higher than that of normal rats (Figure 1C).

HE staining revealed that in normal rats, follicles and lutein cells at different developmental stages, oocytes and corona rays in mature follicles, GCs and thick cell layers in the cutting surface were observed. In the PCOS‐modelled rats, a large number of vesicular dilated follicles were identified, accompanied by loose arrangement of GCs, thinned cell layer, proliferation of cortical cells and a small number of lutein cells (Figure 1D).

During the induction of PCOS rat models, the content of ox‐HDL was detected in serum at the 10th, 20th and 30th of DHEA treatment. It was found that the content of ox‐HDL in the serum of PCOS rats increased gradually with the pathological changes of PCOS, while the content of ox‐HDL in the serum of normal rats did not differ significantly (Figure 1E).

### 3.2 ox‐HDL accelerates endocrine disorders and ovarian damage in rat PCOS models

**FIGURE 2:**
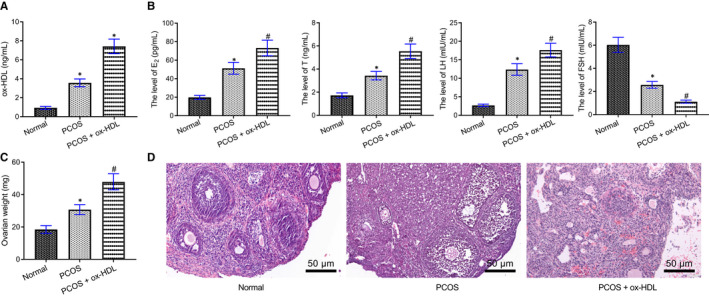
ox‐HDL promotes endocrine disorders and ovarian damage in rats. Rats were injected with normal saline as control, and PCOS rats were untreated or treated with ox‐HDL. A, The level of ox‐HDL in serum of rats detected by ELISA on the 20th day after modelling, * *P* < .05 compared with the normal group. B, Serum E_2_, T, LH and FSH levels in rats detected by ELISA, * *P* < .05 compared with the normal group, # *P* < .05 compared with the PCOS group. C, The weight of ovaries, * *P* < .05 compared with the normal group, # *P* < .05 compared with the PCOS group. D, The histopathological changes of ovarian tissues of rats observed by using HE staining (× 200). Measurement data were expressed as mean ± standard deviation. Unpaired t test was used for data comparison between two groups and one‐way ANOVA was used for data comparison among multiple groups, followed by Tukey's post hoc test. The experiment was repeated 3 times

The expression of ox‐HDL was the highest in the PCOS + ox‐HDL group (Figure 2A). Moreover, the serum E_2_, T and LH levels in PCOS rats were elevated by treatment with ox‐HDL, while FSH level was decreased (Figure 2B). The weight of ovaries of PCOS rats was significantly enhanced by ox‐HDL treatment (Figure 2C).

HE staining (Figure 2D) showed that in normal rats, obvious vesicular dilated follicles and loosely arranged GCs were observed. In the PCOS group, the GC layer decreased, most of them were 3‐4 layers, sheath cells proliferated, and lutein cells decreased. In ox‐HDL‐treated PCOS rats, lutein cells and follicular at different developmental stage decreased, almost no dominant follicle was observed, GCs decreased, and atresia follicle and vesicular expansion follicle were observed.

### 3.3 ox‐HDL promotes GC apoptosis

**FIGURE 3:**
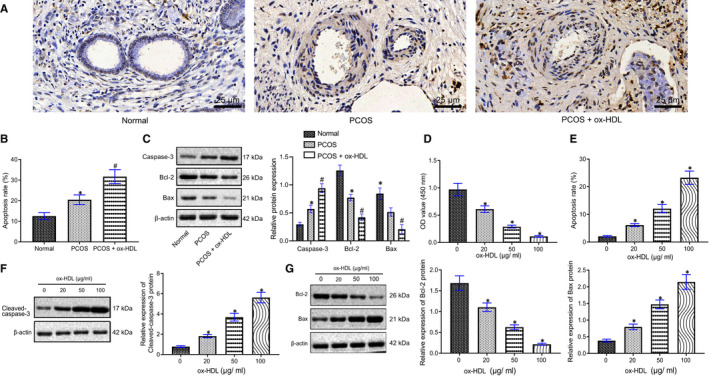
ox‐HDL can promote apoptosis of GCs. A, The apoptosis of GCs in rats of the normal, PCOS, PCOS + ox‐HDL groups detected and quantified by TUNEL staining (× 400). B, The quantification of Panel A. C, The expression of apoptosis‐related proteins cleaved‐caspase‐3, Bcl‐2 and Bax in GCs in rats of the normal, PCOS, PCOS + ox‐HDL groups quantified by Western blot analysis, * *P* < .05 compared with the normal group, #, *P* < .05 compared with the PCOS group. D, The cell viability measured at different concentrations of ox‐HDL (0, 20, 50 and 100 μg/mL) by CCK‐8. E, Cell apoptosis after different treatment detected and quantified by Flow cytometry (Annexin V as x‐axis, PI as y‐axis; upper left quadrant as cell fragments, upper right quadrant as late apoptotic or necrotic cells, lower left quadrant as negative normal cells, lower right quadrant as early apoptotic cells). F, The expression of apoptosis‐related protein caspase‐3 after ox‐HDL treatment determined using Western blot analysis. G, The expression of apoptosis‐related proteins Bcl‐2 and Bax after ox‐HDL treatment determined using Western blot analysis. * *P* < .05 compared with the former group. Measurement data were expressed as mean ± standard deviation. One‐way ANOVA was used for data comparison among multiple groups, followed by Tukey's post hoc test. The experiment was repeated 3 times

TUNEL staining and Western blot analysis manifested that the apoptosis of ovarian GCs in rats was significantly increased after PCOS modelling, accompanied by increase in caspase‐3 and Bax levels and reduction in Bcl‐2 expression, and these trends were further promoted by ox‐HDL treatment (Figure 3A‐C).

CCK‐8 demonstrated that the viability of ovarian GCs was inhibited by ox‐HDL in a concentration gradient manner (Figure 3D). Flow cytometry showed that ox‐HDL promoted apoptosis of ovarian GCs in a concentration‐dependent manner (Figure 3E). Western blot analysis found that cleaved‐caspase‐3 and Bax expression increased, while Bcl‐2 expression decreased in ovarian GCs by ox‐HDL in a concentration‐dependent manner (Figure 3F, G).

### 3.4 Ox‐HDL promotes p65 phosphorylation and miR‐34a expression in GCs

**FIGURE 4:**
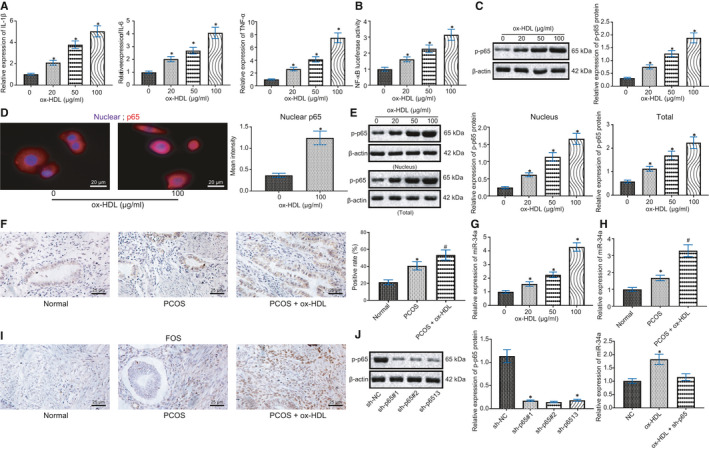
ox‐HDL promotes p65 phosphorylation to up‐regulate miR‐34a expression. A, The mRNA expression of downstream factors of NF‐κB signalling pathway (IL‐1β, IL‐6 and TNFα) in GCs after incubated with different concentrations of ox‐HDL (0, 20, 50 and 100 μg/mL) for 24 h detected by RT‐qPCR. B, NF‐κB activity in GCs by dual luciferase reporter assay. C, The level of p‐p65 in GCs by Western blot analysis. * *P* < .05 compared with the former group. D, Detection and quantification of p65 nuclear translocation in GCs by immunofluorescence (× 500). * *P* < .05 compared with the GCs without ox‐HDL treatment. E, Detection and quantification of p‐p65 in GC in nucleus and GCs, * *P* < .05 compared with the former group. F, The level of p‐p65 in the ovaries of rats by Immunohistochemistry (× 400), * *P* < .05 compared with the normal group, # *P* < .05 compared with the PCOS group. G, miR‐34a expression in GCs detected by RT‐qPCR after incubation with different concentrations of ox‐HDL (0, 50, 100 and 200 μg/mL) for 24 h, * *P* < .05 compared with the former group. H, miR‐34a expression in ovaries of rats by RT‐qPCR, * *P* < .05 compared with the normal group, # *P* < .05 compared with the PCOS group. I, Expression of miR‐34a in ovaries of rats by In situ hybridization (× 400). J, The expression efficiency of sh‐p65 determined by Western blot, and the expression of miR‐34a in GCs after different treatment by RT‐qPCR, * *P* < .05 compared with the sh‐NC or NC group. Measurement data were expressed as mean ± standard deviation. Unpaired t test was used for data comparison between two groups and one‐way ANOVA was used for data comparison among multiple groups, followed by Tukey's post hoc test. The experiment was repeated 3 times

RT‐qPCR presented that the mRNA expression of downstream factors of NF‐κB signalling pathway (IL‐1β, IL‐6 and TNFα) increased by ox‐HDL in a dose‐dependent manner (Figure 4A), which was further confirmed by ELISA results (Supplementary Figure 1). Dual luciferase reporter assay found that NF‐κB activity increased after 24 h incubation with ox‐HDL (Figure 4B). Further Western blot analysis confirmed that ox‐HDL incubation increased p‐p65 (Figure 4C). In addition, immunofluorescence and quantitative analysis of p65 nuclear translocation in GCs showed that ox‐HDL treatment could promote p65 nuclear translocation (Figure 4D). Western blot analysis revealed that ox‐HDL incubation increased the phosphorylation level of S536 site of p65 in nucleus (Figure 4E). Immunohistochemistry depicted that p‐p65 expression in GC nucleus of PCOS rats was significantly increased, which was further augmented by ox‐HDL treatment (Figure 4F).

After incubation with different concentrations of ox‐HDL (0, 50, 100 and 200 μg/mL) for 24 h, RT‐qPCR revealed that miR‐34a expression was increased after ox‐HDL treatment in a dose‐dependent manner (Figure 4G). RT‐qPCR also observed that miR‐34a expression in rats was significantly increased after PCOS modelling, and ox‐HDL treatment further promoted this trend in PCOS rats (Figure 4H). In situ hybridization detection demonstrated the same experimental results in ovarian tissues of rats (Figure 4I).

Subsequently, Western blot analysis results evidently showed the efficiency of sh‐p65. RT‐qPCR showed that ox‐HDL could not promote miR‐34a expression after p65 silencing (Figure 4J).

### 3.5 ox‐HDL inhibits FOS protein expression by p65/miR‐34a axis

**FIGURE 5:**
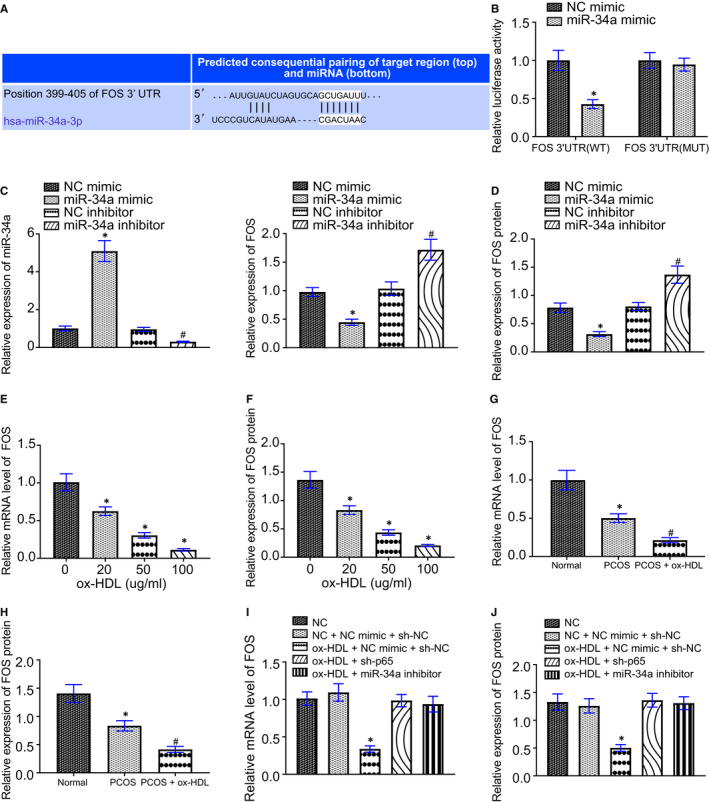
ox‐HDL targets and suppresses FOS protein expression through p65/miR‐34a axis. A, Targetscan prediction of targeting relation between miR‐34a and FOS, and the conservation of FOS target sequence in a variety of animals. B, The effect of miR‐34a on the luciferase activity of 3'UTR of FOS by dual luciferase reporter assay, * *P* < .05 compared with the NC mimic group. C, The level of miR‐34a and mRNA level of FOS in GCs detected by RT‐qPCR. D, Detection and quantification of FOS protein expression in GCs by Western blot analysis, * *P* < .05 compared with the NC mimic group, ^#^ *P* < .05 compared with the NC inhibitor group. E, The expression of FOS in GCs detected by RT‐qPCR, * *P* < .05 compared with the former group. F, Detection and quantification of FOS protein expression in GCs by Western blot analysis, * *P* < .05 compared with the former group. G, Detection of FOS expression in rat ovaries by RT‐qPCR. H, Detection of FOS protein expression in rat ovaries by Western blot analysis. * *P* < .05 compared with the normal group, ^#^ *P* < .05 compared with the PCOS group. I, Expression of FOS in GCs by RT‐qPCR. J, Detection and quantification of FOS protein expression in GCs by Western blot analysis. * *P* < .05 compared with the NC group. Measurement data were expressed as mean ± standard deviation. Unpaired t test was used for data comparison between two groups and one‐way ANOVA was used for data comparison among multiple groups, followed by Tukey's post hoc test. The experiment was repeated 3 times

Targetscan predicted that miR‐34a targeted FOS and that the target sequence was highly conserved in a variety of animals (Figure 5A). Dual luciferase reporter assay showed that miR‐34a mimic significantly inhibited the luciferase activity of FOS 3'UTR‐WT, but had no significant effect on the luciferase activity of FOS 3'UTR‐MUT (Figure 5B). RT‐qPCR and Western blot analysis revealed that miR‐34a mimic diminished but miR‐34a inhibitor augmented FOS expression (Figure 5C, D).

RT‐qPCR and Western blot analysis indicated that FOS expression decreased after ox‐HDL treatment in a dose‐dependent manner (Figure 5E, F). RT‐qPCR and Western blot analysis also displayed that FOS expression in ovarian tissues of rats was decreased after PCOS modelling, which was further reduced by ox‐HDL treatment (Figure 5G, H). In addition, ox‐HDL was incapable of inhibiting FOS expression after treatment with miR‐34a inhibitor or sh‐p65 in GCs (Figure 5I, J).

### 3.6 ox‐HDL promotes GC apoptosis through p65/miR‐34a/FOS axis

**FIGURE 6:**
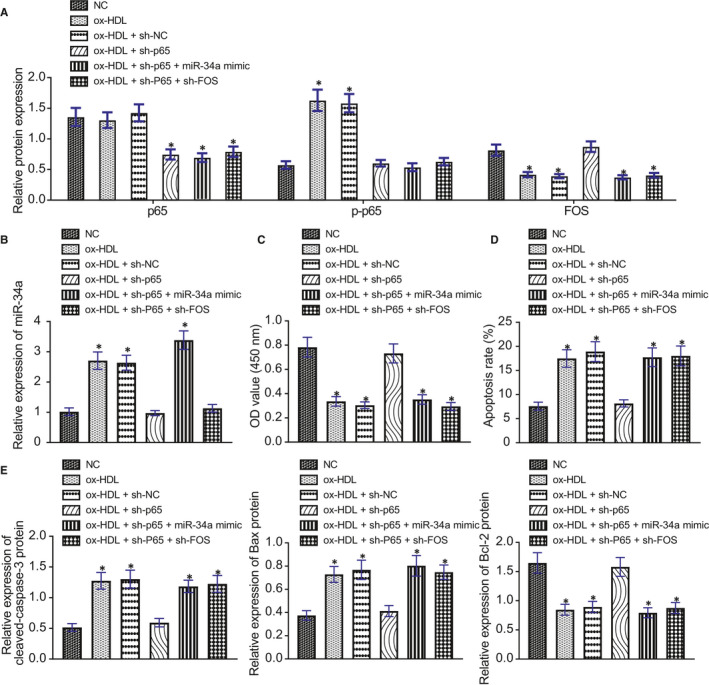
ox‐HDL could promote GC apoptosis through p65/miR‐34a/FOS axis. A, Detection of p65, p‐p65 and FOS expression in GCs by Western blot analysis. B, Expression of miR‐34a in GCs by RT‐qPCR. C, The viability of GCs was detected by CCK‐8. D, The apoptosis of GCs detected and quantified by flow cytometry. E, The expression of apoptosis‐related proteins cleaved‐caspase‐3, Bcl‐2 and Bax in GCs detected and quantified by Western blot analysis. * *P* < .05 compared with the NC group. Measurement data were expressed as mean ± standard deviation. One‐way ANOVA was used for data comparison among multiple groups, followed by Tukey's post hoc test. The experiment was repeated 3 times

After treatment with lentivirus, GCs underwent incubation with ox‐HDL for 24 h. Western blot analysis manifested that p65 expression was significantly reduced in ox‐HDL‐treated GCs by treatment with sh‐p65, sh‐p65 + miR‐34a mimic or sh‐p65 + sh‐FOS versus untreated GCs. Compared with untreated GCs, p‐p65 level markedly rose in ox‐HDL‐treated GCs or ox‐HDL + sh‐NC‐treated GCs. Besides, FOS expression was declined in GCs by treatment with ox‐HDL, ox‐HDL + sh‐NC, ox‐HDL + sh‐p65 + miR‐34a mimic or ox‐HDL + sh‐p65 + sh‐FOS in contrast to untreated GCs (Figure 6A). RT‐qPCR revealed that miR‐34a expression in GCs was elevated as a result of treatment with ox‐HDL, ox‐HDL + sh‐NC or ox‐HDL + sh‐p65 + miR‐34a mimic compared to untreated GCs (Figure 6B).

Subsequently, CCK‐8 (Figure 6C), flow cytometry (Figure 6D) and Western blot analysis (Figure 6E) revealed a decrease in GC viability, while GC apoptosis was increased accompanied by augmented caspase‐3 and Bax levels and declined Bcl‐2 expression after ox‐HDL treatment, which was neutralized by sh‐p65. sh‐p65 + miR‐34a mimic or sh‐p65 + oe‐FOS could not inhibit changes of GC viability, apoptosis and expression of apoptosis‐related proteins induced by ox‐HDL treatment.

### 3.7 ox‐HDL accelerates endocrine disorders and ovarian damage in rats through p65/miR‐34a/FOS axis

**FIGURE 7:**
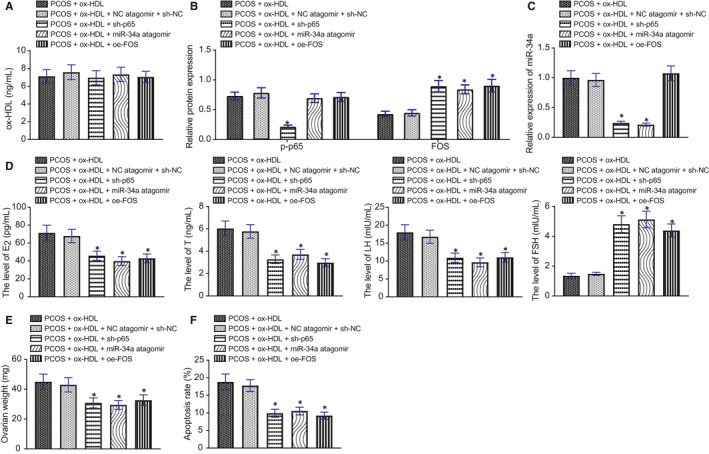
ox‐HDL may accelerate endocrine disorders and ovarian damage in rats through p65/miR‐34a/FOS axis. A, The expression of ox‐HDL in serum of rats detected by ELISA. B, The expression of p‐p65 and FOS in ovarian tissues of rats determined and quantified by Western blot in each group. C, The expression of miR‐34a in ovarian tissues of rats determined and quantified by RT‐qPCR. D. Serum E_2_, T, LH and FSH levels in the serum of rats detected by ELISA. E, The weight of ovaries measured directly. F, The apoptosis of GCs detected using TUNEL staining. * *P* < .05 compared with the NC group. Measurement data were expressed as mean ± standard deviation. One‐way ANOVA was used for data comparison among multiple groups, followed by Tukey's post hoc test. The experiment was repeated 3 times

On the 20th day after modelling, there was no significant difference in serum ox‐HDL content after treatment with different lentiviruses in the presence of PCOS + ox‐HDL (Figure 7A). In the presence of ox‐HDL, p‐p65 expression of PCOS rats was decreased by sh‐p65 but was not affected by NC antagomir + sh‐NC, miR‐34a antagomir or oe‐FOS. In addition, FOS expression of PCOS rats was enhanced by sh‐p65, miR‐34a antagomir or oe‐FOS but was not changed by NC antagomir + sh‐NC in the presence of ox‐HDL (Figure 7B). In the presence of ox‐HDL, miR‐34a expression of PCOS rats was diminished by sh‐p65 or miR‐34a antagomir, while no significant changes were observed with NC antagomir + sh‐NC or oe‐FOS (Figure 7C).

ELISA described that in the presence of PCOS + ox‐HDL, E_2_, T and LH levels were decreased but FSH level was increased in rats after treatment with sh‐p65, miR‐34a antagomir or oe‐FOS (Figure 7D). Next, the weight of ovaries of rats was reduced by treatment with sh‐p65, miR‐34a antagomir or oe‐FOS in the presence of PCOS + ox‐HDL (Figure 7E).

TUNEL staining demonstrated that in the presence of ox‐HDL, apoptosis of GCs in PCOS rats were diminished by treatment with sh‐p65, miR‐34a antagomir or oe‐FOS (Figure 7F).

### 3.8 Ox‐HDL induces p‐p65 and miR‐34a expression, but inhibits FOS expression in normal rats

GCs were treated with ox‐HDL, and the results showed the increased phosphorylation of p65 and thus elevated miR‐34a expression. After normal rats were treated with ox‐HDL, RT‐qPCR and Western blot analysis (Supplementary Figure 3A, B) suggested up‐regulated p‐p65 and miR‐34a but down‐regulated FOS in the isolated GC cells from normal rats. In conclusion, ox‐HDL treatment could elevate p65 or miR‐34a expression, but reduced FOS expression in normal rats.

## 4 DISCUSSION

Polycystic ovary syndrome (PCOS) is a common condition affecting 8% to 13% of reproductive‐aged women.^3^ The key findings from this research highlighted the promoting effect of ox‐HDL in PCOS and provided evidence that accumulation of ox‐HDL contributed to activation of p65 and transcription of miR‐34a, which resulted in the inhibition of FOS expression, thus promoting apoptosis of GCs and accelerating PCOS development.

In line with present findings, the PCOS mice (PCOS mouse model induced by DHEA) also exhibited elevated T and estradiol levels, elevated LH levels and LH/FSH ratio, and decreased ovarian follicular development, increased follicle atresia and cystic follicles, which were consistent with features exhibited in PCOS patients.^14^ In addition, serum levels of E2, T and LH were increased, and levels of FSH were reduced, the proliferation of GCs was reduced and apoptosis was promoted in PCOS‐modelled rat serum.^15^ These findings were suggestive of successful modelling of PCOS. PCOS patients presented with increased HDL secondary to synbiotics supplementation,^5^ and HDL particles were also increased after lipid ingestion.^16^ Furthermore, ox‐HDL would promote EPCs apoptosis and impair EPCs function in disturbed neovascularization in chronic ischaemic disease involved with activation of CD36‐p38 MAPK‐TSP‐1 pathway.^17^

ox‐HDL was found to activate NF‐κB by binding with LOX‐1 on the cell surface, thus promoting the production of reactive oxygen species (ROS) in endothelial cells.^18^ Furthermore, ox‐HDL activates p38 MAPK and NF‐κB pathways, leading to an increase in apoptosis and ROS level, while reducing migration, angiogenesis and cholesterol outflow of EPCs in a dose‐dependent manner.^17^ Similarly, our results revealed that ox‐HDL can result in the activation of NF‐κB p‐p65 in ovarian tissues extracted from PCOS‐modelled rats, promoting miR‐34a expression. In addition, a previous study suggested that IL‐6 or TNF‐α‐activated NF‐κB p65 could bind to miR‐34a promoter and up‐regulate its transcription, which could serve as a novel mechanistic and therapeutic insight into autoimmune disease progression.^8^ Moreover, miR‐34a‐5p has been found to be involved in controlling post‐transcriptomic regulation of the ovulatory process.^19^ miR‐34a has also been shown to promote GC apoptosis in pig ovarian follicles.^9^

Subsequently, Targetscan predicted that miR‐34a targeted FOS expression. Several miRNAs have been identified to be capable of regulating FOS expression in GC apoptosis and proliferation in ovarian GCs that occurs secondary to microcystin‐LR exposure.^20^ It was also observed that c‐FOS might influence ovarian cancer progression through its pro‐apoptotic effect and by altering peritoneal adhesion of OC cells.^21^ Moreover, cold stress resulted in an increase in the number of FOS‐immunoreactive neurons in the locus coeruleus in polycystic ovary in rats.^22^ miR‐421‐5p targeted c‐FOS expression to regulate ovarian growth and development *via* the MAPK signalling pathway in light polluted ovaries obtained from rats.^23^

## 5 CONCLUSION

With the aforementioned findings taken into consideration, our study validated the hypothesis that ox‐HDL activated NF‐κB p65 to promote miR‐34a expression, resulting in a decrease in FOS expression to promote apoptosis of GCs, leading to an overall enhancement of ongoing of PCOS (Supplementary Figure 2). Thus, the ox‐HDL/NF‐κB p65/miR‐34a/FOS axis might serve as a potential therapeutic strategy for PCOS. However, future studies centred around specimens obtained from PCOS‐diagnosed patients are recommended to thoroughly understand the ox‐HDL/NF‐κB p65/miR‐34a/FOS axis. The specific mechanism involving the ox‐HDL/NF‐κB p65/miR‐34a/FOS axis in cellular network alterations also requires further explorations as it has been scarcely studied.

## CONFLICT OF INTEREST

The authors declare no competing interest.

## AUTHORS CONTRIBUTION

Lu Wang: Conceptualization; methodology. Hongjuan Li: Formal analysis; investigation. Xiaoke Tang: Data curation; resources. Yupei Yang: Project administration; writing‐review and editing. Yuancui Xiang: Methodology; visualization. Hui Zhang: Supervision; software. Yali Wang: Conceptualization, funding acquisition; writing‐original draft.

## Supporting information

Fig S1

Fig S2

Fig S3

## Data Availability

Research data are not shared.
