# Supplementary figures and images for: Oxidized high‐density lipoprotein enhances endocrine disorders and ovarian damage in rats
Source: J Cell Mol Med. 2021 Aug 4;25(17):8115–26. doi: 10.1111/jcmm.16197 (PMC8419193; doi:10.1111/jcmm.16197)

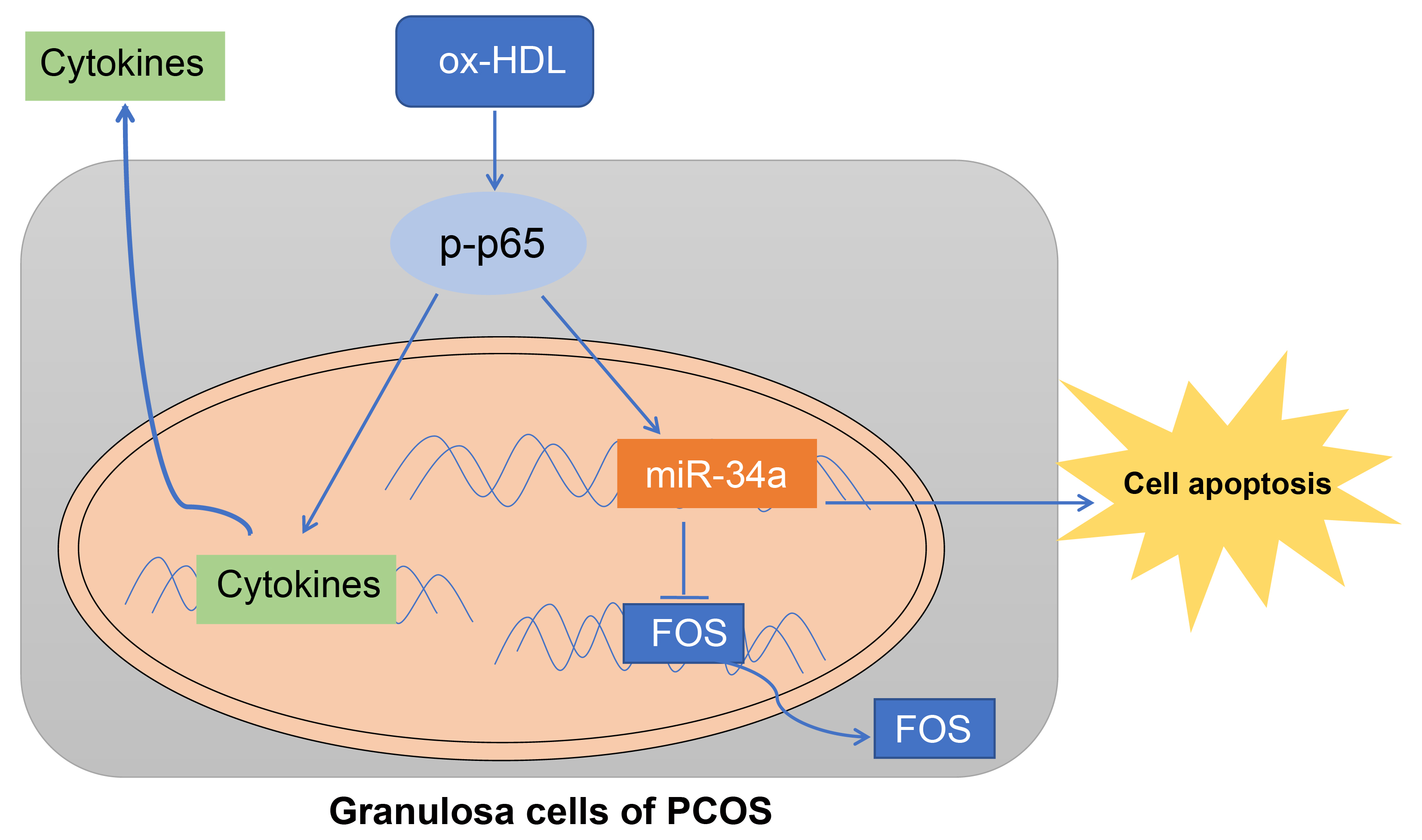

Supplement: Supplementary file 2 — Fig S2 [file JCMM-25-8115-s003.jpg]
